# Differential Function of a Novel Population of the CD19+CD24hiCD38hi Bregs in Psoriasis and Multiple Myeloma

**DOI:** 10.3390/cells10020411

**Published:** 2021-02-16

**Authors:** Joanna Bartosińska, Joanna Purkot, Agnieszka Karczmarczyk, Michał Chojnacki, Joanna Zaleska, Paulina Własiuk, Norbert Grząśko, Marta Morawska, Adam Walter-Croneck, Lidia Usnarska-Zubkiewicz, Patrycja Zielińska, Krzysztof Jamroziak, Małgorzata Kowal, Dorota Krasowska, Grażyna Chodorowska, Krzysztof Giannopoulos

**Affiliations:** 1 Department of Cosmetology and Aestetic Medicine, Medical University of Lublin, 20-093 Lublin, Poland; joanna.bartosinska@umlub.pl; 2 Experimental Hematooncology Department, Medical University of Lublin, 20-093 Lublin, Poland; joannapurkot@gmail.com (J.P.); agnieszka.piechnik@umlub.pl (A.K.); michalchojnacki@umlub.pl (M.C.); malazam150@gmail.com (J.Z.); paulina.wlasiuk@umlub.pl (P.W.); norbert.grzasko@umlub.pl (N.G.); martamorawska@umlub.pl (M.M.); 3 Department of Hematooncology and Bone Marrow Transplantation, Medical University of Lublin, 20-080 Lublin, Poland; adam.walter-croneck@umlub.pl; 4 Department of Hematology, Blood Neoplasms and Bone Marrow Transplantation, Wroclaw Medical University, 50-367 Wroclaw, Poland; lidia.usnarska-zubkiewicz@umed.wroc.pl; 5 Department of Hematology and Bone Marrow Transplantation, Medical University of Silesia, 40-032 Katowice, Poland; paula.zielinska@doctoral.uj.edu.pl; 6 Department of Hematology, Institute of Hematology and Transfusion Medicine, 02-776 Warszawa, Poland; krzysztof.jamroziak@wp.pl; 7 Department of Dermatology, Venereology and Pediatric Dermatology, Medical University of Lublin, 20-080 Lublin, Poland; kowalma71@o2.pl (M.K.); dor.krasowska@gmail.com (D.K.); grazyna.chodorowska@umlub.pl (G.C.)

**Keywords:** psoriasis, multiple myeloma, Bregs, interleukin 10

## Abstract

Psoriasis (Ps), an autoimmune disease, and multiple myeloma (MM), a blood neoplasm, are characterized by immune dysregulation resulting from the imbalance between the effector and regulatory cells, including B regulatory (Breg) lymphocytes. Peripheral blood samples from 80 Ps patients, 17 relapsed/refractory MM patients before and after daratumumab (anti-CD38 monoclonal antibody) treatment, 23 healthy volunteers (HVs), and bone marrow samples from 59 MM patients were used in the study. Bregs were determined by flow cytometry using CD19, CD24, and CD38. Intracellular production of interleukin-10 (IL-10) was assessed by flow cytometry after CD40L, LPS, and CpG stimulation. IL-10 serum or plasma concentrations were tested using ELISA method. The percentage of CD19+CD24hiCD38hi Bregs was not different whereas the production of IL-10 in Bregs was significantly higher in Ps patients in comparison with HVs. The percentage of CD19+CD24hiCD38hi Bregs in MM patients was significantly higher than in HVs (*p* < 0.0001). The percentage of CD19+CD24hiCD38hi Bregs was significantly higher in MM patients with the ISS stage I (*p* = 0.0233) while IL-10 production in Bregs was significantly higher in ISS stage III (p = 0.0165). IL-10 serum or plasma concentration was significantly higher in Ps and MM patients when compared to HVs (*p* < 0.0001). Following the treatment with daratumumab the percentages of CD19+CD24hiCD38hi Bregs significantly decreased (*p* < 0.0003). Here, in the two opposite immune conditions, despite the differences in percentages of Bregs in Ps and MM we have identified some similarities in the IL-10 producing Bregs. Effective treatment of daratumumab besides the anti-myeloma effect was accompanied by the eradication of Bregs.

## 1. Introduction

The regulatory B cells (Bregs) are essential contributors to the pathogenesis of inflammatory and autoimmune conditions as well as a neoplastic disease [1].

Bregs are known to mediate inflammation and maintain homeostasis and peripheral immune tolerance mainly via secretion of suppressive cytokines, such as interleukin-10 (IL-10), interleukin-35 (IL-35), and the transforming growth factor-β (TGF-β), as well as via expression of inhibitory molecules, such as the programmed cell death ligand 1 (PD-L1) [1,2]. The level of IL-10 production by Bregs is indicative of their regulatory functions; thus, the Bregs’ cell subsets producing IL-10, ranging from early immature transitional B cells (CD24hiCD38hi) to highly differentiated plasmablasts (CD27intCD38+) and plasma cells, are collectively referred to as the B10 cells [1,3,4]. As Bregs have not been assigned a definitive phenotype yet, the production of IL-10 is still the best functional hallmark or phenotypic marker to identify Bregs.

The role of Bregs in modulating the immune response consists of compromising the T cells’ immunity and facilitation of their conversion into the regulatory T cells (Tregs), which is executed through the communication of CD40 on the Bregs’ surface with CD40L on the effector T cells, thereby leading to the T cells’ death and reduced response to the autoantigen [1]. Blair et al. [3] found that within the peripheral blood of healthy individuals CD40 stimulation leads to the generation of IL-10-producing B cells, the highest fraction of which are the CD19+CD24hiCD38hi B cells. Upon the CD40 stimulation, Bregs, due to producing IL-10, can suppress Th1 and Th2 production and differentiation, generation, and response of Th17, and secretion of pro-inflammatory cytokines. They are also able to inhibit antigen presentation by mononuclear cells, macrophages, and dendritic cells (DCs) [3].

Therefore, any imbalance in the number or function in favor of the immune effector cells may contribute to the development of autoimmune and autoinflammatory conditions, whereas upregulation of the regulatory cells, i.e., regulatory T cells (Tregs) and also Bregs, may accompany tumorigenesis [1,5,6].

Psoriasis (Ps), an autoinflammatory disease in which activation of antigen-presenting cells (APCs), Th1, and Th17 cells and increased production of proinflammatory cytokines (IFNγ, TNF-alpha, IL-23/IL-17 axis) leads to impaired immune tolerance, is an exemplification of the presented immune imbalance [7]. It is known that the functionality of the regulatory cells in Ps is compromised, among other things, by decreased expressions of certain molecules involved in the intercellular contact, i.e., B7(CD86)/cytotoxic T-lymphocyte–associated antigen-4 CTLA-4, CD40/CD40L, PD-1/PD-L1. Since in Ps the regulatory cells, such as Tregs, are deficient in preventing the T cell activation and maintaining adequate control of immune responses, the use of immune checkpoint inhibitors, i.e., anti-PD-1 (nivolumab, pembrolizumab), anti-PD1L (durvalumab), and anti-CTLA-4 (ipilimumab, tremelimumab), in malignant tumors and hematologic neoplasm may cause autoimmune/autoinflammatory side effects, including Ps [8,9,10,11,12,13,14].

Present in the malignant microenvironment of multiple myeloma (MM), the inflammatory component promotes proliferation and survival of malignant cells, angiogenesis, and adaptive immunity, which has an overwhelming impact on the functionality of the regulatory cells [15]. It is well established that in MM immune abnormalities not only in the B cells but also in other immune cells are observed, including natural killer (NK), T cells, and DCs. Moreover, Tregs and myeloid-derived suppressor cells (MDSCs) may be associated with the disease progression [16,17], but once the effector T cells’ exhaustion and a suppressive bone marrow microenvironment implicated in the MM progression are established, the MM immunotherapy becomes ineffective and MM progresses into relapsed/refractory MM (RRMM) [18]. Therefore, targeting CD38+ cells, i.e., plasma cells and non-plasma cells responsible for immunosuppression (e.g., proliferating Tregs) with daratumumab (DARA), an anti-CD38 monoclonal human IgG1 kappa antibody, may be an effective strategy in the treatment of MM patients [19].

Both Ps and MM are characterized by immune dysregulation which results from the imbalance between the effector and regulatory cells, and the role of Bregs is still awaiting further clarification.

The aim of the study was an analysis of the CD19+CD24hiCD38hi Bregs’ frequency in the MM patients and Ps patients as well as the comparison of CD19+CD24hiCD38hi Bregs’ frequency in the patients with RRMM before and after daratumumab treatment. The study also aimed at assessing the CD19+CD24hiCD38hi Bregs’ functionality on the basis of IL-10 intracellular production and its serum or plasma concentrations in comparison to healthy volunteers.

## 2. Materials and Methods

### 2.1. The Study Groups

Peripheral blood mononuclear cells (PBMCs) were isolated from 80 psoriatic patients who had not received any anti-psoriatic treatment for at least 6 months prior to the recruitment into the study. PBMCs were also collected from 17 RRMM patients before and after daratumumab treatment and 23 healthy volunteers (HVs). Bone marrow mononuclear cells (BMMCs) were obtained from 59 MM patients at the time of diagnosis. PBMC of MM patients were collected from 9 patients at the time of diagnosis.

The severity of Ps was assessed with the use of Psoriasis Area and Severity Index (PASI).

All of the MM patients met the International Myeloma Working Group (IMWG) Criteria for the Diagnosis of MM and stage of the disease was graded using the International Staging System (ISS).

RRMM patients were qualified to the DaraCup patient program in Poland if they met criteria described previously [20]. Patients received daratumumab in monotherapy with dose 16 mg/kg, intravenously, once weekly for 8 weeks, next, once every 2 weeks for 16 weeks, and then once every 4 weeks thereafter until the progression of the disease or unacceptable toxicity. PBMCs were collected and analyzed before treatment and after 5 cycles of daratumumab treatment.

Informed consent was obtained from each individual. The study was approved by the local ethics committee of the Medical University of Lublin. The demographic data of the patients and HVs as well as the clinical characteristics of the patients are shown in Table 1.

### 2.2. Blood and Bone Marrow Sampling

Peripheral blood (PB) samples from the Ps patients, RRMM patients before and after daratumumab treatment, and control HVs, as well as bone marrow (BM) samples from the MM patients obtained at the time of diagnosis, were collected in EDTA tubes. PBMCs and BMMCs were isolated using Biocoll density gradient centrifugation (Biochrom, Berlin, Germany). Serum samples were collected from the Ps patients whereas plasma samples were collected from the MM patients, RRMM patients before and after daratumumab treatment, and control HVs by centrifuging at 2200 rpm for 15 min and immediately storing in −80 °C.

### 2.3. Bregs Phenotypic Analysis and Detection of Intracellular IL-10 Production

For evaluation of Bregs and their ability to produce intracellular IL-10, 48-h cell cultures and flow cytometry analysis were performed. PBMCs of Ps and MM patients at the time of diagnosis and HVs were suspended at the concentration of 2 × 10^6^/mL in RPMI1640 medium (Biochrom, Germany) with 10% FBS (Biochrom, Germany) in the presence of mitogens–1 µg/mL CD40L (R&D Systems, Minneapolis, MN), 10 µg/mL LPS (Sigma-Aldrich, St. Louis, MO, USA), and 10 µg/mL CpG ODN 2006 (Invivogen, San Diego, CA, USA). For the last 5 h, cells were treated with 50 ng/mL PMA (Sigma), 1 µg/mL ionomycin (Sigma), and 5 µg/mL, brefeldin A (BFA, Sigma). The surviving cells were enumerated by trypan blue exclusion. After 48 h cells were harvested and stained for surface markers with anti-CD19 Pe-Cy7 (BD Biosciences, San Jose, CA, USA), anti-CD24 APC (Beckman Coulter, Marseille, France), and anti-CD38 FITC (BD Biosciences, San Jose, CA, USA) monoclonal antibodies for 20 min in RT in the dark. For analysis of intracellular marker, cells were washed and fixed with Reagent 1 from IntraPrep Leukocytic Permeabilization Reagent (Beckman Coulter, Marseille, France) at RT for 15 min and washed with PBS, then cells were permeabilized with Reagent 2 (Beckman Coulter, Marseille, France) for 5 min in RT and stained with 20 µl of anti-IL10 PE (BD Biosciences, San Jose, CA, USA) antibodies at RT for 20 min according to staining protocol [21,22]. Next, cells were washed with PBS and the production of IL-10 by Bregs was analyzed by FACSuite on BD FACS Lyric Flow Cytometer (BD Biosciences, San Jose, CA, USA). Results were compared to negative control cells without antibodies, and Fluorescence Minus One (FMO) control in the absence of anti-IL-10 PE monoclonal antibody.

### 2.4. Enzyme-Linked Immunosorbent Assay (ELISA)

The concentration of serum or plasma IL-10 in individual subjects was tested using human IL-10 Quantikine ELISA Kit (R&D Systems, cat. No D1000B) according to the manufacturer’s protocol. Plates were scanned by Victor 3 Plate Reader (Perkin Elmer, Waltham, MA, USA).

### 2.5. Statistical Analyses

Statistical analyses were performed using GraphPad Prism 5 (La Jolla, California, United States). The Mann–Whitney *U-test* and Kruskal–Wallis test were used to evaluate the differences between the subgroups. The correlations of variables were computed with the Spearman rank correlation coefficient. To evaluate the differences in values before and after drug treatment, Wilcoxon matched-pairs signed-rank test was implemented. FACS data were analyzed with FACSuite software (BD Biosciences, San Jose, CA, USA). Percentages of Bregs and mean fluorescence intensities (MFI) were described as medians. Statistical significance was defined as a *p* value of less than 0.05.

## 3. Results

### 3.1. CD19+CD24hiCD38hi Bregs Are Increased in BMMCs of MM Patients, Whereas They Are Similar in PBMCs of Ps and MM Patients as Compared to HV

**Figure 1:**
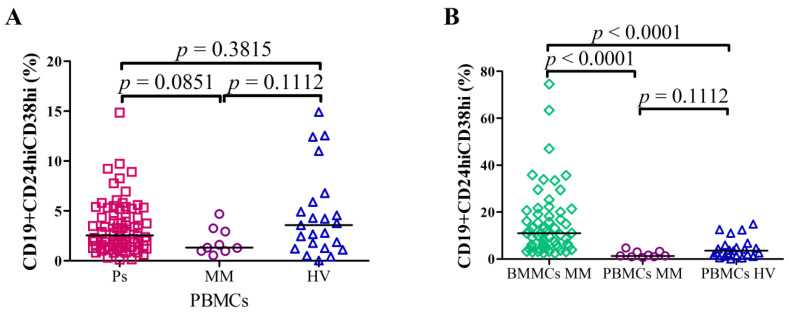
The percentages of CD19+CD24hiCD38hi Bregs in (**A**) PBMCs of Ps patients (N = 80) vs. PBMCs of MM patients (N = 9) vs. PBMCs of HVs (N = 23) and (**B**) BMMCs of MM patients (N = 59) vs. PBMCs of MM patients (N = 9) vs. PBMCs of HVs (N = 23). There were no significant differences in CD19+CD24hiCD38hi Bregs levels in PBMCs between Ps patients and HVs as well as MM patients (**A**). The percentage of Bregs was increased in BMMCs of MM patients in comparison with PBMCs of HVs (*p* < 0.0001) as well as in comparison with PBMCs of MM patients (*p* < 0.0001).

Flow cytometry was used to analyze the frequency of Bregs after short term cell cultures in Ps as well as MM patients. The percentage of CD19+CD24hiCD38hi Bregs in PBMCs of Ps patients (median = 2.55%; range = 0.1%–14.86%) was not significantly different in comparison with HVs (median = 3.58%; range = 0.03%–14.92%; *p* = 0.3815) and PBMCs of MM (median = 1.32%; range = 0.54%–4.69%; *p* = 0.1112). There was no significant difference between the levels of CD19+CD24hiCD38hi Bregs in PBMCs of MM patients and in PBMCs of Ps patients (*p* = 0.0851) (Figure 1A).

Interestingly, the percentage of CD19+CD24hiCD38hi Bregs in BMMCs of MM patients was significantly higher: the median = 10.08% (range = 2.46%–74.58%) as compared with PBMCs of HVs: median = 3.58% (range = 0.03%–14.92%; *p* < 0.0001) as well as with PBMCs of MM: median = 1.32% (range = 0.54%–4.69%; *p* < 0.0001) (Figure 1B). Example of flow cytometric analysis of Bregs from BMMCs (A) and PBMCs (B) is displayed in Figure S2. Differential representation of CD19 subpopulations based on CD24 and CD38 expression in analyzed groups of patients (Figure S1.)

### 3.2. Higher Serum or Plasma IL-10 Concentrations in Ps Patients and MM Patients as Compared to HVs

**Figure 2:**
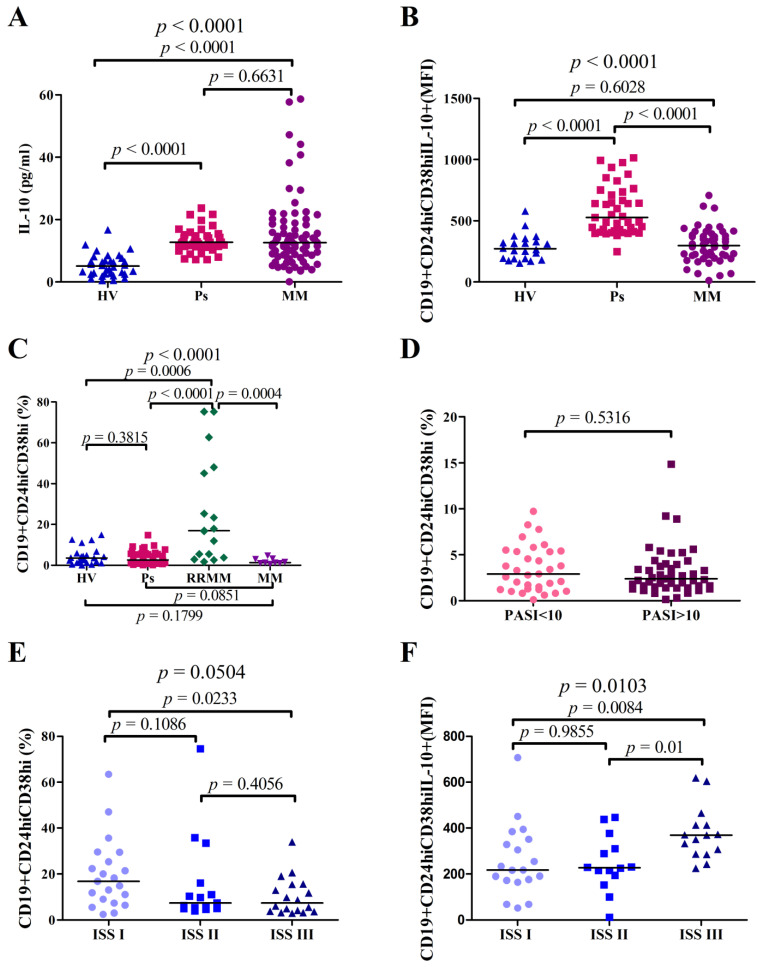
Differences in interleukin-10 (IL-10) serum or plasma levels as well as percentages of CD19+CD24hiCD38hi Bregs and IL-10 production in PBMCs of Ps, BMMCs of MM, PBMCs of RRMM patients, and PBMCs of HVs. Analyses between two groups were performed using Mann–Whitney *U-test* while topmost p values were obtained from Kruskal-Wallis test. (**A**) Significantly higher IL-10 serum levels in Ps patients vs. IL-10 plasma levels of HVs (*p* < 0.0001) and plasma of MM patients vs. HVs (*p* < 0.0001). (**B**) Flow cytometry analysis of mean fluorescence intensities (MFI) of CD19+CD24hiCD38hi IL-10+ in HVs, Ps, and MM patients demonstrated increased production of IL-10 in Ps patients in comparison with HVs (*p* < 0.0001). Cells were surface-stained with anti-CD19-Pe-Cy7, anti-CD24-APC, anti-CD38-FITC, and intracellularly with IL10-PE antibodies. (**C**) Chart showing significantly elevated percentages of CD19+CD24hiCD38hi Bregs in PBMCs of RRMM patients in comparison with HVs (*p* = 0.0006) as well as vs. PBMCs of MM (*p* = 0.0004) and no difference between PBMCs of Ps patients vs. HVs. (**D**) The percentages of CD19+CD24hiCD38hi according to PASI stage system in Ps patients. (**E**) The percentages of Breg cells in the different clinical ISS stages in MM patients showed a significant decrease of Bregs level in ISS stage III in comparison with ISS stage I (*p* = 0.0233). (**F**) Assessment of the CD19+CD24hiCD38hi IL-10 MFI of MM patients according to ISS stages demonstrated significantly elevated intensity in ISS stage III vs. ISS stage I (*p* = 0.0084) and ISS stage II (*p* = 0.01).

The IL-10 serum concentration was significantly higher in Ps patients: median = 12.73 pg/mL (range = 7.160 pg/mL–23.71 pg/mL) when compared to plasma of HVs: median = 5.076 pg/mL (range = 0.4538 pg/mL–16.72 pg/mL; *p* < 0.0001). It was also significantly higher in plasma of MM patients when compared to HVs: the median of IL-10 in MM patients = 12.64 pg/mL (range = 0.09257 pg/mL–58.66 pg/mL), whereas in HVs the median = 5.076 pg/mL (range = 0.4538 pg/mL–16.72 pg/mL; *p* < 0.0001) (Figure 2A).

We compared IL-10 levels in Ps patients due to the severity of the disease evaluated by PASI stage system and we found no significant difference in IL-10 levels between the groups (*p* = 0.411). IL-10 levels were compared in MM patients divided into groups according to the ISS staging system and we also found no significant difference (*p* = 0.9192).

### 3.3. IL-10 Production in Bregs Is Higher in Ps Patients Than in HVs and MM Patients

**Figure 3:**
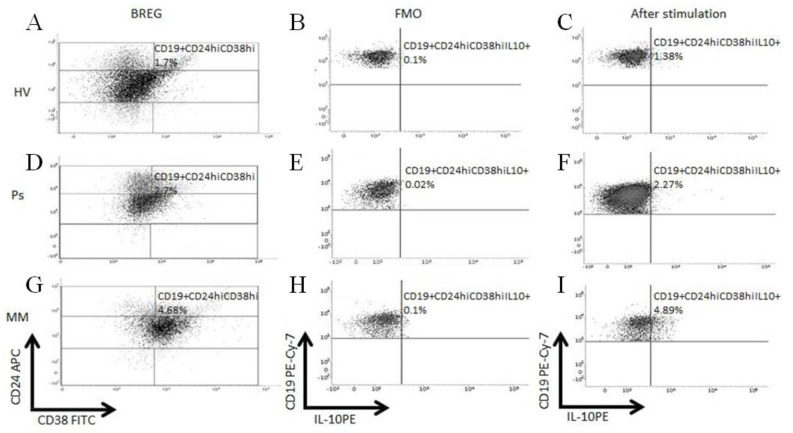
Flow cytometry analysis of intracellular expression of interleukin-10 (IL-10) in Breg cells. Scatter dot plots illustrated examples of Breg analyses in PBMCs of HVs, Ps, and BMMCs of MM patients (**A**,**D**,**G**), the Fluorescence Minus One Control (FMO) controls was used as negative control for IL-10 expression (**B**,**E**,**H**) and dot plots demonstrating percentages of IL-10 producing cells after incubation with mitogens (**C**,**F**,**I**). Cells were surface-stained with anti-CD19 Pe-Cy7, anti-CD24-APC, anti-CD38-FITC, and intracellularly with anti-IL10-PE antibodies.

To assess the functionality of Bregs in Ps and MM patients, the intracellular IL-10 production was measured by flow cytometry (Figure 3).

The mean fluorescence intensity (MFI) of the IL-10 expression in CD19+CD24hiCD38hi Bregs was significantly higher in Ps patients: median = 527 (range = 248–1014) as compared with HVs: median = 271 (range = 154–579; *p* < 0.0001), and patients with MM: median = 297 (range = 12–707; *p* < 0.0001). Statistical analysis revealed no differences between medians of MFI of IL-10 expression in CD19+CD24hiCD38hi Bregs of MM patients vs. HVs (*p* = 0.6028) (Figure 2B).

The percentages of CD19+CD24hiCD38hi Bregs in PBMCs of RRMM patients were significantly higher in comparison to HVs (median = 3.58%; range = 0%–14.92%; *p* = 0.0006), Ps patients (median = 2.55%; range = 0.1%–14.86%; *p* < 0.0001), and PBMCs of MM patients (median = 1.32%; range = 0.54%-4.69%; *p* = 0.0004) (Figure 2C).

### 3.4. Correlations between the Percentage of CD19+CD24hiCD38hi Bregs and PASI in Ps Patients as well as ISS Stage in MM Patients

Due to discovered elevated levels of Breg cells in Ps as well as MM patients in comparison with HVs we analyzed an association between Breg expression levels and psoriasis subtype as well as severity of these diseases. The patients with an early onset of psoriasis (type I) had higher percentages of CD19+CD24hiCD38hi B cells in comparison to the patients with a late onset of psoriasis (type II) (*p* = 0.0281) There was a weak reverse correlation between age and the percentage of CD19+CD24hiCD38hi Bregs.

In Ps patients, the percentage of CD19+CD24hiCD38hi Bregs did not correlate with the severity of the disease calculated by PASI (R = 0.08, *p* = 0.4334). There was no statistical difference in percentage of CD19+CD24hiCD38hi Bregs between patients with mild psoriasis PASI < 10: median = 2.9 (range = 0.1%–9.72%) and with moderate to severe psoriasis PASI ≥ 10 median = 2.37 (range = 0.14%–14.86%; *p* = 0.5316) (Figure 2D).

In MM patients, the percentage of CD19+CD24hiCD38hi Bregs was not significantly different, but there was a tendency between the three ISS stages of the disease (*p* = 0.0504). Furthermore, the percentage of CD19+CD24hiCD38hi Bregs was significantly higher in MM patients with the ISS stage I than in those with the ISS stage III (*p* = 0.0233) (Figure 2E). However, the MFI of IL-10 expression in CD19+CD24hiCD38hi Bregs was significantly higher in patients with III stage of MM (*p* = 0.0084) (Figure 2F).

### 3.5. Decreased CD19+CD24hiCD38hi Bregs in RRMM Patients after Daratumumab Treatment

**Figure 4:**
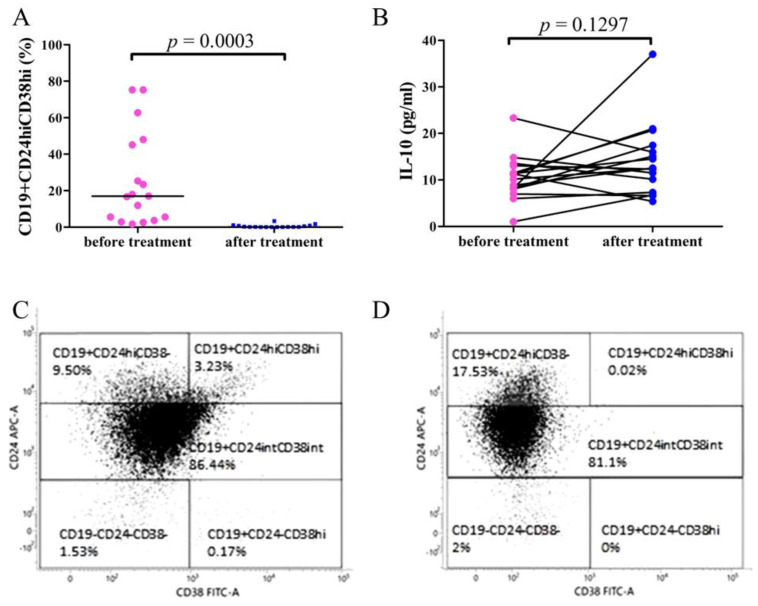
The frequencies of CD19+CD24hiCD38hi Breg cells (**A**) and IL-10 concentration (**B**) in PBMCs of RRMM patients before and after 5 cycles of daratumumab treatment. (**A**) Flow cytometry analysis demonstrated significantly increased expression of Bregs in RRMM patients before the treatment with daratumumab compared to samples obtained after 5 cycles of the therapy (*p* = 0.0003). (**B**) ELISA test results showed no significant differences of IL-10 levels in RRMM patients after daratumumab treatment (*p* = 0.1297). The representative dot plots of Breg subpopulations showing CD19+CD24hiCD38hi Breg population before (**C**) and after daratumumab treatment (**D**). Cells were stained with surface-stained with anti-CD19 Pe-Cy7, anti-CD24-APC, anti-CD38-FITC, and intracellularly with IL10-PE antibodies.

PBMCs of 17 patients with RRMM (12 female and 5 male, median age 59 (range: 47–82 years)) was assessed by flow cytometry before and after 5 cycles of daratumumab treatment. Statistical analyses demonstrated that the percentage of CD19+CD24hiCD38hi Bregs was significantly lower after daratumumab treatment: median = 0.11% (range: 0%–3.35%) in comparison with the patients’ samples before the treatment: median = 10.08% (range: 2.46%–74.58%; *p* < 0.0003) (Figure 4A).

The flow cytometry dot plots of CD19+CD24hiCD38hi Bregs of the representative RRMM patient before and after daratumumab treatment are shown in Figure 4C,D.

### 3.6. Comparison of IL-10 Plasma Concentration in RRMM Patients before and after Daratumumab Treatment

The assessment of plasma concentration of IL-10 in RRMM patients showed no significant difference in samples obtained before the treatment with daratumumab: median = 10.63 pg/mL (range 1.003 pg/mL–23.32 pg/mL) and after 5 cycles of therapy: median = 12.49 pg/mL (range 5.387 pg/mL–37. pg/mL; *p* = 0.1297) (Figure 4B).

## 4. Discussion

Physiologically, the balance between the immune effector cells and immunosuppressive regulatory cells prevents the development of immune dysregulation. Bregs, which can downregulate excessive immune and inflammatory responses, comprise several phenotypically distinct B-cell lineages which are identified by production of cytokines, i.e., IL-10, IL-35, and TGF-beta. It is still unclear if the Bregs producing the IL-10, IL-35, and TGF-beta cytokines are overlapping or if they represent different stages of the B cells. Therefore, apart from the action of the immunomodulatory cytokine IL-10, the other mechanisms of Bregs functioning include production of TGF-beta that downregulates the Th1 immunity, promotes Tregs expansion as well as IL-35 production which inhibits the Th1/Th17 cells, and promotes Tregs. This proves a significant immunosuppressive function of Il-35 and its role in promoting a pro-tumor phenotype [21]. Hayashi et al. [22] observed that in MM patients the plasma cells and BM stromal cells produce higher concentrations of TGF-beta compared with the plasma cells from HVs.

Since the regulatory cells’ imbalance is observed in autoimmune/autoinflammatory and neoplastic processes, in the present study an attempt was made to investigate the CD19+CD24hiCD38hi Bregs’ frequency in two selected conditions, i.e., Ps and MM. In the former, the mononuclear cells obtained from the peripheral blood were investigated, while in the latter, as the bone marrow is the compartment of active disease, the bone marrow mononuclear cells were collected to be studied.

Even though reports on Bregs in Ps and MM are scarce, Hayashi et al. [23] reported that IL-10-producing Bregs were decreased in Ps patients, which, according to them, was suggestive of the B10 cells’ functionality impairment in this disease. However, the same authors also noticed that the frequency of CD19+CD24hiCD38hi Bregs, which are identified as progenitors of B10 cells, was significantly higher in the Ps patients than in controls. They observed that after treatment with immunosuppressants, i.e., infliximab, ustekinumab, and cyclosporine A, the frequency of B10 cells increased, while the frequency of progenitors of B10 cells decreased. Mavropoulos et al. [24], who investigated CD19+CD24hiCD38hi Bregs in both Ps and psoriatic arthritis patients (PsA), reported that the IL-10 producing Bregs are impaired in both Ps and PsA and inversely correlated with IL-17- and IFNgamma-producing T cells. Mavropoulos et al. [24] also observed that the IL-10(+) Bregs were inversely correlated with the severity of Ps. They suggested that Bregs could participate in the immune dysregulation of Ps and PsA. Quite recently, the same authors investigated the effect of apremilast (a phosphodiesterase 4 inhibitor) in Ps and PsA on Bregs and found out that apremilast induced increased Bregs, which was associated with a decrease in the Th1 cells and IFNgamma- and IL-17-producing NKT cells [25].

An observation that Ps could be induced in the course of lymphoid cancers treated with rituximab, an anti-CD20 antibody, suggested the participation of Bregs in the Ps pathogenesis [26]. Moreover, in a mouse model of imiquimod-induced psoriasis, a more severe skin inflammation observed in the CD19−/− mice in comparison to the wild type mice was indicative of the IL-10(+) CD1d(high)CD5(+) Bregs’ role in suppressing the psoriasis-like inflammation [27].

In our study, the percentage of CD19+CD24hiCD38hi Bregs in Ps patients was similar to that of healthy controls and did not correlate with the severity of the disease. Since Bregs are a small group of cells, in order to assess their ability to produce IL-10 after stimulation, we measured the mean fluorescence intensity (MFI) of IL-10 production. Although the percentage of CD19+CD24hiCD38hi Bregs was unchanged in the studied Ps patients, the MFI of IL-10 production in the CD19+CD24hiCD38hi Bregs was significantly higher in them in comparison with HVs. This could mean that Bregs in Ps patients may have a robust regulatory capacity and high potential to produce IL-10 and they attempt to downregulate the ongoing inflammation. However, they prove to be ineffective in attenuating the disease. Therefore, their functionality needs to be strengthened by effective therapeutic agents. This is in agreement with the studies of Mavropoulos et al. [25] and Hayshi et al. [23] who observed increased Bregs producing IL-10 following the use of apremilast and immunosuppressants in Ps patients.

While B cell suppression of the T effector cells responses during chronic inflammation may be beneficial in Ps, this same immune-modulatory activity may allow malignant cells to survive and proliferate in the chronically inflamed environment. It is known that Bregs reduce cellular responses and promote tumor growth and metastases due to the Treg cells’ generation and suppression of effector T cells and NK cells’ responses when the antitumor effect of T cells is simultaneously suppressed by IL-10 produced by the B10 cells [28].

Zhang et al. [29], in their group of newly diagnosed multiple myeloma (NDMM) patients, detected frequencies of Bregs within the CD19+ B cells in the peripheral blood which were not significantly different from those observed in the healthy subjects (5.44 ± 1.97% versus 4.56 ± 0.86%). However, the authors revealed significantly higher Bregs in the bone marrow (BM) than in PB of NDMM patients. Moreover, Bregs in BM were significantly higher in the patients with NDMM than in those on maintenance therapy after response to treatment with elotuzumab. The same authors observed that the MM cells reduce apoptosis and promote survival of Bregs, which will mediate immunosuppression by the production of IL-10. Zhang et al. [29] investigated the function of BM-derived Bregs of NDMM patients and observed an increased IL-10 production after stimulation of the Bregs.

In our study, we found that the percentage of the CD19+CD24hiCD38hi Bregs in BMMCs was significantly higher in patients with MM as compared with the percentage of the CD19+CD24hiCD38hi Bregs in PBMCs in HVs, which was in agreement with the immunosuppressive properties of Bregs reported by Zhang et al. [29]. The percentage of CD19+CD24hiCD38hi Bregs was significantly higher in ISS stage I of the disease. We observed that even though the BM of MM patients was enriched with the CD19+CD24hiCD38hi Bregs, the median MFI of IL-10 in the CD19+CD24hiCD38hi Bregs in MM patients and HVs was not different. This could suggest that IL-10 production by a single CD19+CD24hiCD38hi Breg in MM patients does not differ from IL-10 production by the same type of cell in HVs. However, the increased amount (percentage) of the CD19+CD24hiCD38hi Bregs in MM patients may lead to an increase in IL-10 production. Interestingly, IL-10 production in CD19+CD24hiCD38hi Bregs correlated with the severity of MM and was significantly higher in the patients with ISS stage III of the disease. Our study results might suggest that with the increased stage of the disease the percentage of CD19+CD24hiCD38hi Bregs tended to decrease while their ability to produce IL-10, i.e., MFI of IL-10 in CD19+CD24hiCD38hi Bregs, tended to increase.

IL-10 produced not only by Bregs but also by Tregs, monocytes, some of the NK cells, macrophages, DCs, innate lymphoid cells (ILCs), mast cells, and eosinophils, inhibits the activity of DCs and macrophages, and suppresses the production of Th1, Th2, and Th17 [1,30,31,32]. In a double-blind study, McInnes et al. [33] observed that in PsA patients administered IL-10 subcutaneously for 28 days their psoriatic skin lesions improved and they also had a decreased number of T cells and macrophage infiltrations in the synovial tissue. Even though the anti-inflammatory effect of IL-10 is unquestionable and its effectiveness in Ps therapy has been confirmed, the study results on its serum concentrations in Ps patients are not conclusive. Some authors, e.g., Takahashi et al. [34] and Jacob et al. [35], observed that the levels of IL-10 were decreased in the Ps patients. In contrast, Borska et al. [36] and Deeva et al. [37] found that the IL-10 serum concentration was higher in Ps patients in comparison with the control groups. In the study of Hayashi et al. [23], the serum IL-10 levels in Ps patients and HVs did not differ significantly. However, a meta-analysis performed by Dowlatshahi et al. [38] revealed a small positive but not statistically significant difference in IL-10 levels between Ps patients and controls, independently of the age, sex, PASI, or psoriasis type. Therefore, these controversial study results cannot unequivocally confirm IL-10 as a reliable biomarker for psoriasis.

In the present study, since our Ps patients were severely ill, i.e., they had high PASI (mean, 12.05), their IL-10 serum concentration was found to be significantly higher in comparison to IL-10 plasma concentration in HVs. In our Ps patients, IL-10 serum concentration did not correlate with PASI. Despite this, it seems that increased IL-10 serum concentration was not sufficient to be able to counteract the proinflammatory action of the Th1 and Th17 cells and cause immunosuppression.

In MM, IL-10 is known to play an immunosuppressive role and favor the progress of this disease. IL-10 could augment the proliferation of B cells and affect their differentiation into the plasma cells in MM [39,40].

More recent studies have confirmed that IL-10 acts as a growth factor for the MM cells and that its elevated serum levels could be associated with a more advanced stage of the disease [41,42]. Wang et al. [43] reported higher IL-10 serum levels in MM patients in comparison with healthy controls and they suggested that increased IL-10 levels correlated significantly with worse clinical-pathological features. In another study, by Alexandrakis et al. [41], higher IL-10 serum concentrations in MM patients compared with controls were reported. Similarly, Pappa et al. [44], observed that elevated amounts of IL-10 correlated positively with advanced stages of MM. Thus, the IL-10 levels could be easily evaluated in MM patients and could serve as a remarkable prognostic factor.

In our study, the IL-10 plasma concentration was significantly higher in MM patients when compared to HVs, which confirms the findings of the aforementioned studies. However, in our patients, IL-10 plasma did not significantly differ between the ISS stages of MM.

Scientific research into IL-10 production is expected to give grounds for the implementation of novel therapeutic options in MM. Daratumumab, a human IgG1 antibody that targets CD38 and has a direct antitumor and immunomodulatory activity, attaches to the CD38 protein found on the surface of myeloma cells, thereby enabling the immune system to target and kill it. With daratumumab, an overall response was observed in 42–71% of patients and occurred mostly during the first infusion, which is indicative of daratumumab’s ability to counteract the immunosuppressive activity of the CD38 cells [45].

In our study, we investigated the percentage of Bregs also during daratumumab monotherapy; here, the study material was collected from the peripheral blood. Following the treatment with daratumumab, the percentages of CD19+CD24hiCD38hi Bregs were significantly decreased. Interestingly, the percentages of CD19+CD24hiCD38hi Bregs decreased rapidly just after the first cycle of the treatment. However, the plasma concentration of IL-10 in the studied RRMM patients was not significantly different before and after the daratumumab treatment. The decreased Bregs’ percentage and unchanged concentration of plasma IL-10 are suggestive of other sources of IL-10 in MM patients. Nevertheless, the results of our study indicate that the treatment of RRMM with daratumumab causes an immediate decrease in CD19+CD24hiCD38hi Bregs.

## 5. Conclusions

In conclusion, in this study, we have characterized the Breg frequencies and function in Ps and MM. In Ps CD19+CD24hiCD38hi Bregs frequencies were unchanged but their IL-10 production was increased, while in MM CD19+CD24hiCD38hi Bregs frequencies did not change in different ISS stages but their IL-10-production potential increased in ISS III stage. Moreover, the study revealed that daratumumab might not only target directly MM cells but also Bregs with no indirect impact on IL-10 production.

## Figures and Tables

**Table 1:** Demographic and clinical characteristics of the studied groups (MM, RRMM, Ps, HVs).

Characteristics	MM (N = 59)	RRMM (N = 17)	Ps (N = 80)	HVs (N = 23)
Age (years), median (range)	68 (41–84)	59 (47–82)	47 (18–77)	28 (18–58)
Sex				
Female, n	26	12	14	10
Male, n	33	5	66	13
ISS disease stage, n (%)		n/a	n/a	n/a
I	21 (38.9)			
II	15 (27.8)			
III	18 (33.3)			
	5 n/a			
PASI, median (range)	n/a	n/a	12.05 (2.7–49.4)	n/a

n/a, not applicable; MM, multiple myeloma; Ps, psoriasis; RRMM, relapsed/refractory multiple myeloma; HVs, healthy volunteers; ISS, the International Staging System; PASI, Psoriasis Area and Severity Index.

## Data Availability

The data presented in this study are available on request from the corresponding author. The data are not publicly available due to privacy.

## Supplementary Materials

The following are available online at https://www.mdpi.com/2073-4409/10/2/411/s1, Figure S1: The comparison of percentages of other Breg subpopulations in HV, MM, Psoriatic, and daratumumab patients, Figure S2: Gating strategy for evaluation of Breg cells in bone marrow and peripheral blood.
