# Supplementary material for: Differential Function of a Novel Population of the CD19+CD24hiCD38hi Bregs in Psoriasis and Multiple Myeloma
Source: Cells. 2021 Feb 16;10(2):411. doi: 10.3390/cells10020411 (PMC7920433; doi:10.3390/cells10020411)
Supplement: Supplementary file 1 [file cells-10-00411-s001.pdf]

Differential Function of a Novel Population of the CD19+CD24hiCD38hi Bregs in Psoriasis and Multiple Myeloma

Supplementary Materials:

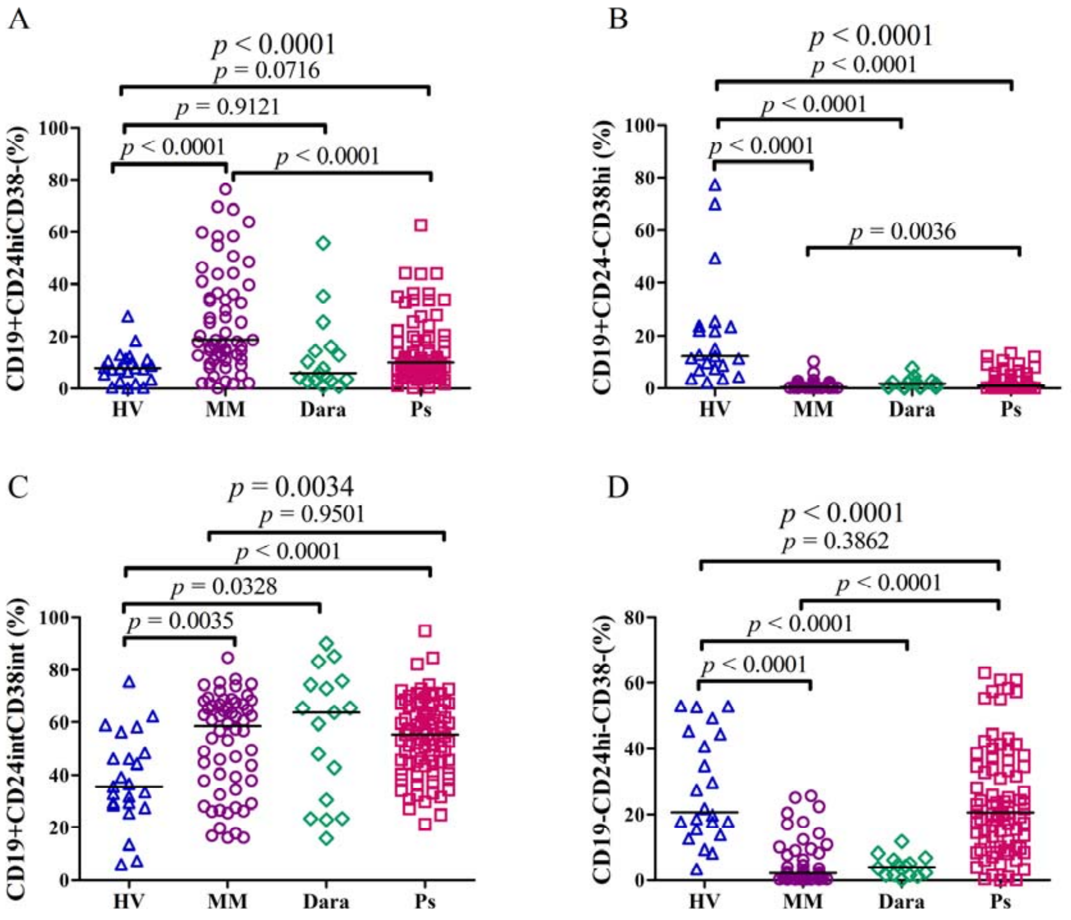

Figure S1. The comparison of percentages of other Breg subpopulations in HV, MM, Psoriatic and daratumumab patients: CD19+CD24hiCD38- (**A**), CD19+CD24-CD38hi (**B**), CD19+CD24intCD38int (**C**), CD19+CD24hi-CD38- (**D**).

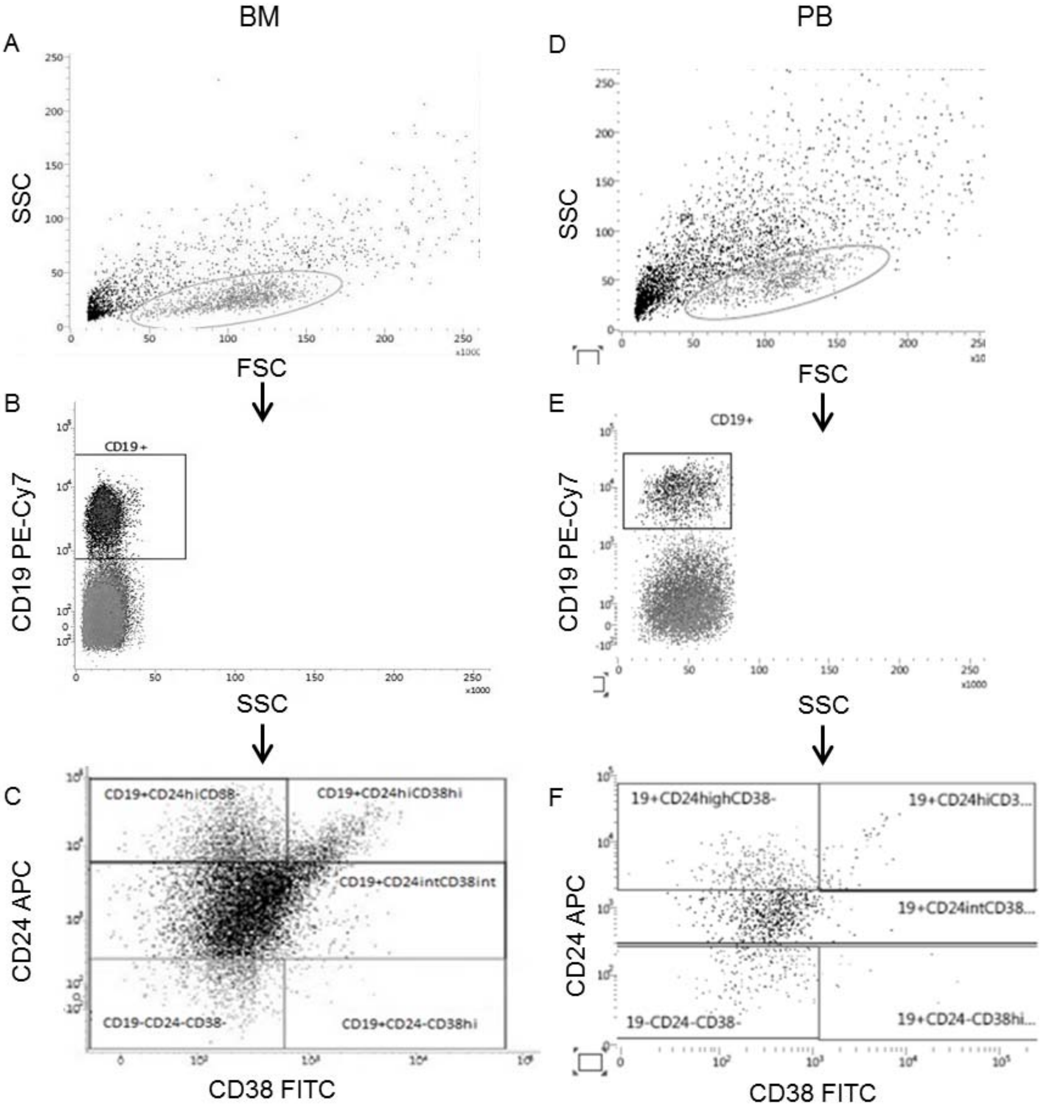

Figure S2. Gating strategy for evaluation of Breg cells in bone marrow (**A–C**) and peripheral blood (**D–F**). B cells gated from lymphocytes on SSC/FSC scatter dot plot (**A**,**D**), were defined as CD19+ lymphocytes. Within CD19+ B cells gate (**B**,**E**), CD24hiCD38hi cells were defined as CD24hiCD38hi Bregs (**C**,**F**).
